# 2-(Octa­decyl­sulfan­yl)-1,3-thia­zole

**DOI:** 10.1107/S2414314620001704

**Published:** 2020-02-11

**Authors:** Joseph E. Muller II, Laura R. Osborn, Joseph R. Traver, Patrick C. Hillesheim, Matthias Zeller, Arsalan Mirjafari

**Affiliations:** a Department of Chemistry and Physics, Florida Gulf Coast University, 10501 FGCU Blvd. South, Fort Myers, FL, 33965, USA; b Ave Maria University, Department of Chemistry and Physics, 5050 Ave Maria Blvd, Ave Maria FL, 34142, USA; c Purdue University, Department of Chemistry, 560 Oval Drive, West Lafayette, Indiana USA, 47907, USA; Howard University, USA

**Keywords:** crystal structure, thia­zole, alkyl chains

## Abstract

The title compound, C_21_H_39_NS_2_, crystallizes with two mol­ecules in the asymmetric unit, both having a linear 18-carbon alkyl chain bound through a thio­ether group. No π–π stacking or hydrogen bonding is observed. The orientation of the alkyl chains facilitates inter­molecular inter­actions between te chains. The structure is metrically ortho­rhom­bic but crystallizes in the monoclinic space group *P*2_1_ and was found to be twinned by pseudomerohedry (emulating ortho­rhom­bic symmetry) and by inversion. The twin factions refined to 0.37 (4), 0.13 (4), 0.31 (5), and 0.19 (4).

## Abstract 2

The title compound crystallizes with a linear alkyl chain bound to the sulfur atom at the 2-position of the thia­zole ring.

## Graphical abstract

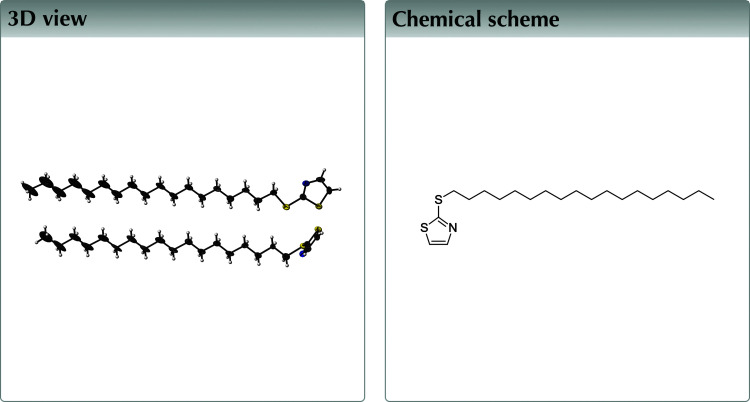

## Structure description

**Figure 1:**
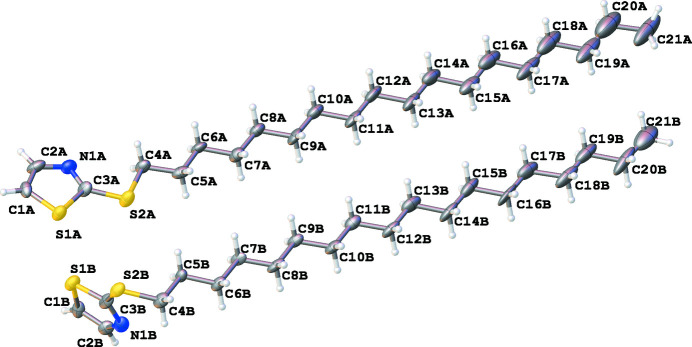
The title compound shown with 50% probability ellipsoids.

**Figure 2:**
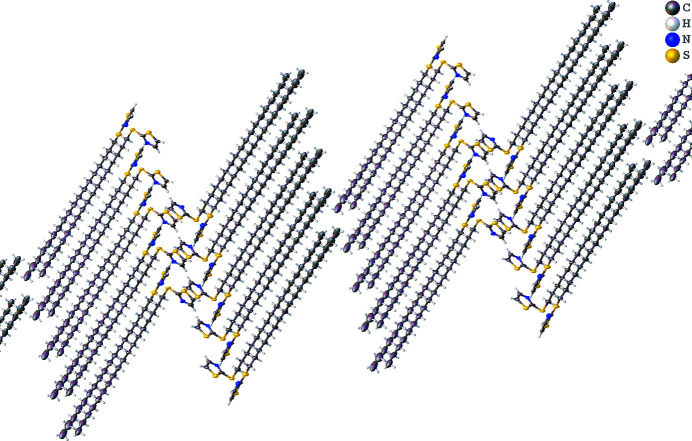
Packing diagram for the title compound depicting the alkyl chain inter­actions between mol­ecules.

The title compound (Fig. 1 ▸) exhibits no notable hydrogen bonding or π–π inter­actions. It appears that inter­actions involving atoms of the alkyl chains form the majority of the inter­molecular inter­actions [*D*⋯*A* = 3.730 (10) to 3.974 (11) Å]. There are two independent mol­ecular units found in the structure, indicated by the atom label suffixes *A* and *B*. While the majority of the two mol­ecules exhibit similar geometrical features, such as a linear alkyl chain, the two mol­ecules differ in the C3—S2—C4—C5 torsion angles [177.9 (7)° in mol­ecule *A* and 70.6 (8)° in mol­ecule *B*. From the packing diagram (Fig. 2 ▸), it appears that mol­ecule *B* adopts this torsion angle to facilitate the alkyl-chain inter­actions while avoiding any repulsive inter­actions with the thia­zole ring of the adjacent mol­ecule *A*.

For the synthesis and applications of alkyl­ated thia­zoles, see: Iwasaki *et al.*, (2016 ▸). For an example of alkyl­ated thia­zoles as metal ligands, see: Artem’ev *et al.* (2018 ▸). For similarly alkyl­ated complexes as ionic liquids, see: Nestor *et al.* (2017 ▸) and O’Brien *et al.*(2016 ▸).

## Synthesis and crystallization

A 250 ml round-bottom flask, oven dried, was paired with a Teflon-coated magnetic stir bar. 2-Mercapto­thia­zole (1.004 g, 1 equiv.) and 1-bromo­octa­decane (2.861 g, 1 equiv.) were dissolved into 150 ml of aceto­nitrile in the 250 ml round bottom flask, which was attached to a water-jacketed reflux condenser and placed into an oil bath. The hot plate was set to 82°C with stirring on and ran for 48 h, after which it was left to cool to room temperature. The solvent was then removed under reduced pressure and a white crystalline solid formed in high yield (92%).

The solid product was dissolved in boiling aceto­nitrile and laboratory parafilm was used to cover the vial, with one hole prodded at the top. Colorless crystals of the product formed over 12 d.

^1^H NMR (400 MHz, chloro­form-*d*) δ 7.65–7.64 (*m*, 1H), 7.19 (*q*, *J* = 1.6 Hz, 1H), 3.19 (*t*, *J* = 7.3 Hz, 2H), 1.77–1.70 (*m*, 2H), 1.45–1.38 (*m*, 2H), 1.24 (*s*, 28H), 0.88–0.85 (*m*, 3H)

^13^C NMR (101 MHz, chloro­form-*d*) δ 142.8, 118.7, 77.4, 77.1, 76.8, 34.7, 32.0, 29.8, 29.7, 29.6, 29.5, 29.3, 29.2, 28.8, 22.8, 14.2

## Refinement

Crystal data, data collection and structure refinement details are summarized in Table 1 ▸. The structure is metrically ortho­rhom­bic but crystallizes in the monoclinic space group *P*2_1_. Initial attempts to solve the structure in various ortho­rhom­bic space groups failed. A closer inspection of diffraction images showed the peaks to be a bit asymmetric, but they were not obviously split. Unit-cell angles were indecisive. Reflection statistics (*XPREP*; Sheldrick, 2008 ▸) indicated a high *R*_sym_ value for ortho­rhom­bic and for two of the three possible monoclinic settings (> 1/5). The third monoclinic option had a low *R*_sym_ (0.05). After relaxation of the default thresholds for maximum intensity for systematically absent reflections, *XPREP* indicated a 2_1_ screw axis, but was indecisive regarding the presence of glide planes because of twin overlaps. Solution attempts in *P*2_1_ in this monoclinic setting were able to localize some of the alkyl chains. The addition of a twin transformation matrix (1 0 0 0 −1 0 0 0 −1) (Rotax within *WinGX*; Farrugia, 2012 ▸) and iterative refinements allowed for the assignment of the remaining atoms from difference density maps. The initial Flack parameter indicated the presence of inversion twinning, and in the final model the structure was refined as four component twinned by pseudo-merohedry (emulating ortho­rhom­bic symmetry) and by inversion. Twin fractions refined to 0.37 (4), 0.13 (4), 0.31 (5) and 0.19 (4). The outer ends of the C18 alkyl chains are ill defined because of large thermal libration and/or ill-defined disorder. The outermost C—C bond distances in the two mol­ecules were restrained to be similar (e.s.d. = 0.02 Å), and a rigid bond restraint (RIGU, e.s.d. = 0.004 Å^2^) was applied for the four outermost two carbon atoms of each mol­ecule.

## Supplementary Material

Crystal structure: contains datablock(s) I, global. DOI: 10.1107/S2414314620001704/bv4027sup1.cif

Structure factors: contains datablock(s) I. DOI: 10.1107/S2414314620001704/bv4027Isup2.hkl

Supporting information file. DOI: 10.1107/S2414314620001704/bv4027Isup3.cml

CCDC reference: 1982241

Additional supporting information:  crystallographic information; 3D view; checkCIF report

## Figures and Tables

**Table 1:** Experimental details

Crystal data	
Chemical formula	C_21_H_39_NS_2_
*M*_r_	369.65
Crystal system, space group	Monoclinic, *P*2_1_
Temperature (K)	150
*a*, *b*, *c* (Å)	5.5457 (6), 9.1108 (10), 43.511 (6)
β (°)	90.376 (5)
*V* (Å^3^)	2198.4 (4)
*Z*	4
Radiation type	Cu *K*α
μ (mm^−1^)	2.19
Crystal size (mm)	0.16 × 0.14 × 0.02

Data collection	
Diffractometer	Bruker AXS D8 Quest CMOS diffractometer with PhotonII charge-integrating pixel array detector (CPAD)
Absorption correction	Multi-scan (*SADABS*; Krause *et al.*, 2015 ▸)
*T*_min_, *T*_max_	0.082, 0.226
No. of measured, independent and observed [*I* > 2σ(*I*)] reflections	20191, 8140, 7233
*R*_int_	0.113
(sin θ/λ)_max_ (Å^−1^)	0.620

Refinement	
*R*[*F* ^2^ > 2σ(*F* ^2^)], *wR*(*F* ^2^), *S*	0.097, 0.263, 1.05
No. of reflections	8140
No. of parameters	438
No. of restraints	44
H-atom treatment	H-atom parameters constrained
Δρ_max_, Δρ_min_ (e Å^−3^)	0.98, −0.75
Absolute structure	Twinning involves inversion, so Flack parameter cannot be determined

Computer programs: *APEX3* and *SAINT* (Bruker, 2018 ▸), *SHELXS97* (Sheldrick, 2008 ▸), *SHELXL2018* (Sheldrick, 2015 ▸, 2018 ▸) and *SHELXLE* (Hübschle *et al.*, 2011 ▸), *OLEX2* (Dolomanov *et al.*, 2009 ▸), *Mercury* (Macrae *et al.*, 2020 ▸), *publCIF* (Westrip, 2010 ▸) and *enCIFer* (Allen *et al.*, 2004 ▸).

## full crystallographic data

### Crystal data

**Table:**

C_21_H_39_NS_2_	*F*(000) = 816
*M**_r_* = 369.65	*D*_x_ = 1.117 Mg m^−^^3^
Monoclinic, *P*2_1_	Cu *K*α radiation, λ = 1.54178 Å
*a* = 5.5457 (6) Å	Cell parameters from 9695 reflections
*b* = 9.1108 (10) Å	θ = 3.0–73.0°
*c* = 43.511 (6) Å	µ = 2.19 mm^−^^1^
β = 90.376 (5)°	*T* = 150 K
*V* = 2198.4 (4) Å^3^	Plate, colourless
*Z* = 4	0.16 × 0.14 × 0.02 mm

### Data collection

**Table:**

Bruker AXS D8 Quest CMOS diffractometer with PhotonII charge-integrating pixel array detector (CPAD)	8140 independent reflections
Radiation source: I-mu-S microsource X-ray tube	7233 reflections with *I* > 2σ(*I*)
Laterally graded multilayer (Goebel) mirror monochromator	*R*_int_ = 0.113
Detector resolution: 7.4074 pixels mm^-1^	θ_max_ = 73.0°, θ_min_ = 2.0°
ω and phi scans	*h* = −5→6
Absorption correction: multi-scan (SADABS; Krause *et al.*, 2015)	*k* = −10→11
*T*_min_ = 0.082, *T*_max_ = 0.226	*l* = −53→53
20191 measured reflections	

### Refinement

**Table:**

Refinement on *F*^2^	Secondary atom site location: difference Fourier map
Least-squares matrix: full	Hydrogen site location: inferred from neighbouring sites
*R*[*F*^2^ > 2σ(*F*^2^)] = 0.097	H-atom parameters constrained
*wR*(*F*^2^) = 0.263	*w* = 1/[σ^2^(*F*_o_^2^) + (0.1736*P*)^2^] where *P* = (*F*_o_^2^ + 2*F*_c_^2^)/3
*S* = 1.05	(Δ/σ)_max_ < 0.001
8140 reflections	Δρ_max_ = 0.98 e Å^−^^3^
438 parameters	Δρ_min_ = −0.75 e Å^−^^3^
44 restraints	Absolute structure: Twinning involves inversion, so Flack parameter cannot be determined
Primary atom site location: structure-invariant direct methods	

### Special details

**Table:**

Geometry. All esds (except the esd in the dihedral angle between two l.s. planes) are estimated using the full covariance matrix. The cell esds are taken into account individually in the estimation of esds in distances, angles and torsion angles; correlations between esds in cell parameters are only used when they are defined by crystal symmetry. An approximate (isotropic) treatment of cell esds is used for estimating esds involving l.s. planes.
Refinement. The structure is metrically orthorhombic but crystallizes in P21. It was found to be twinned by pseudo-merohedry (emulating orthorhombic symmetry) and by inversion and was refined as a 4-component inversion twin. Twin factions refined to 0.37 (4), 0.13 (4), 0.31 (5) and 0.19 (4). The outer ends of the C18 alkyl chain is ill defined due to large thermal libration and/or ill defined disorder. The outermost C-C bond distance in the two molecules was restrained to be similar, and a rigid bond restraint (RIGU) was applied for the four outermost carbon atoms of each molecule. C—H bond distances were constrained to 0.95 Å for thiazole C—H moieties, 0.99 Å for methylene CH_2_ and 0.98 Å for methyl CH_3_ moieties. *U*_iso_(H) values were set to 1.5 times *U*_eq_(C) for methyl groups, and 1.2 times *U*_eq_(C) CH and CH_2_ groups.

### Fractional atomic coordinates and isotropic or equivalent isotropic displacement parameters (Å^2^)

**Table:**

	*x*	*y*	*z*	*U*_iso_*/*U*_eq_	
N1A	−0.9102 (18)	0.9632 (9)	0.08345 (19)	0.0389 (19)	
C1A	−1.2274 (17)	0.8909 (13)	0.05318 (19)	0.035 (2)	
H1A	−1.359748	0.902576	0.039414	0.042*	
S1A	−1.1325 (5)	0.7269 (3)	0.06812 (6)	0.0400 (6)	
C2A	−1.086 (2)	1.0024 (11)	0.0639 (3)	0.044 (2)	
H2A	−1.110693	1.101489	0.057795	0.052*	
S2A	−0.7221 (5)	0.7202 (3)	0.11213 (7)	0.0445 (6)	
C3A	−0.9098 (18)	0.8239 (11)	0.0887 (2)	0.035 (2)	
C4A	−0.5488 (18)	0.8633 (11)	0.1303 (2)	0.036 (2)	
H4AA	−0.454522	0.918792	0.114926	0.043*	
H4AB	−0.655094	0.932302	0.141395	0.043*	
C5A	−0.380 (2)	0.7814 (11)	0.1529 (3)	0.040 (2)	
H5AA	−0.479374	0.723997	0.167394	0.049*	
H5AB	−0.279545	0.711484	0.141175	0.049*	
C6A	−0.221 (2)	0.8817 (11)	0.1705 (2)	0.037 (2)	
H6AA	−0.320040	0.951837	0.182357	0.045*	
H6AB	−0.119161	0.938528	0.156183	0.045*	
C7A	−0.058 (2)	0.7939 (12)	0.1927 (3)	0.042 (2)	
H7AA	−0.161900	0.734007	0.206281	0.050*	
H7AB	0.042393	0.725816	0.180595	0.050*	
C8A	0.104 (2)	0.8881 (11)	0.2124 (2)	0.040 (2)	
H8AA	0.003657	0.958254	0.224072	0.048*	
H8AB	0.211172	0.945739	0.198867	0.048*	
C9A	0.258 (2)	0.8008 (13)	0.2348 (3)	0.046 (2)	
H9AA	0.357113	0.730238	0.223130	0.055*	
H9AB	0.150297	0.743610	0.248404	0.055*	
C10A	0.423 (2)	0.8955 (12)	0.2546 (3)	0.048 (3)	
H10A	0.323170	0.964711	0.266598	0.057*	
H10B	0.528011	0.954173	0.240992	0.057*	
C11A	0.581 (2)	0.8063 (13)	0.2767 (3)	0.050 (3)	
H11A	0.475798	0.746010	0.290008	0.060*	
H11B	0.683071	0.738580	0.264673	0.060*	
C12A	0.739 (3)	0.8992 (13)	0.2965 (3)	0.060 (3)	
H12A	0.636006	0.964899	0.308931	0.072*	
H12B	0.840013	0.961604	0.283162	0.072*	
C13A	0.906 (3)	0.8103 (15)	0.3185 (3)	0.062 (4)	
H13A	0.804874	0.746109	0.331494	0.075*	
H13B	1.011296	0.746216	0.306033	0.075*	
C14A	1.056 (3)	0.9007 (14)	0.3383 (3)	0.064 (4)	
H14A	0.950936	0.963980	0.350929	0.077*	
H14B	1.156295	0.965510	0.325312	0.077*	
C15A	1.226 (3)	0.8098 (16)	0.3601 (3)	0.069 (4)	
H15A	1.126217	0.743174	0.372773	0.083*	
H15B	1.334243	0.748473	0.347510	0.083*	
C16A	1.371 (4)	0.9013 (17)	0.3804 (4)	0.083 (6)	
H16A	1.262449	0.961754	0.393178	0.099*	
H16B	1.468878	0.968876	0.367737	0.099*	
C17A	1.546 (4)	0.810 (2)	0.4023 (4)	0.101 (6)	
H17A	1.447828	0.741971	0.414744	0.121*	
H17B	1.655128	0.750604	0.389467	0.121*	
C18A	1.695 (4)	0.903 (2)	0.4235 (4)	0.109 (7)	
H18A	1.584091	0.960429	0.436584	0.131*	
H18B	1.787191	0.973481	0.410935	0.131*	
C19A	1.880 (5)	0.817 (2)	0.4455 (5)	0.115 (7)	
H19A	1.788400	0.745451	0.457819	0.138*	
H19B	1.993042	0.761172	0.432417	0.138*	
C20A	2.020 (5)	0.907 (3)	0.4661 (6)	0.138 (9)	
H20A	1.905486	0.965017	0.478641	0.166*	
H20B	2.112387	0.977688	0.453576	0.166*	
C21A	2.203 (5)	0.829 (3)	0.4890 (5)	0.152 (10)	
H21A	2.365633	0.831955	0.480288	0.228*	
H21B	2.154139	0.727159	0.492139	0.228*	
H21C	2.203401	0.880998	0.508784	0.228*	
N1B	−0.3610 (15)	0.3613 (9)	0.03120 (17)	0.0332 (16)	
C1B	−0.3453 (17)	0.5615 (11)	−0.0031 (2)	0.034 (2)	
H1B	−0.289999	0.619919	−0.019707	0.041*	
S1B	−0.6079 (5)	0.5923 (3)	0.01858 (6)	0.0389 (5)	
C2B	−0.2370 (18)	0.4401 (11)	0.0081 (2)	0.034 (2)	
H2B	−0.084246	0.408798	0.000816	0.041*	
S2B	−0.7610 (4)	0.3686 (3)	0.06669 (6)	0.0345 (5)	
C3B	−0.5590 (17)	0.4307 (11)	0.0392 (2)	0.038 (2)	
C4B	−0.5670 (18)	0.2595 (13)	0.0909 (3)	0.042 (3)	
H4BA	−0.665925	0.200980	0.105217	0.050*	
H4BB	−0.473032	0.190771	0.078025	0.050*	
C5B	−0.3916 (16)	0.3598 (10)	0.10946 (19)	0.0269 (17)	
H5BA	−0.288999	0.415915	0.095109	0.032*	
H5BB	−0.485597	0.430746	0.121769	0.032*	
C6B	−0.2336 (18)	0.2684 (10)	0.1306 (2)	0.034 (2)	
H6BA	−0.135299	0.200076	0.118203	0.040*	
H6BB	−0.336775	0.209091	0.144346	0.040*	
C7B	−0.0677 (15)	0.3648 (11)	0.1499 (2)	0.0301 (17)	
H7BA	0.034383	0.423721	0.136028	0.036*	
H7BB	−0.167491	0.433812	0.161990	0.036*	
C8B	0.094 (2)	0.2804 (11)	0.1718 (3)	0.039 (2)	
H8BA	−0.008060	0.221157	0.185669	0.047*	
H8BB	0.194445	0.211769	0.159752	0.047*	
C9B	0.2563 (18)	0.3774 (11)	0.1909 (2)	0.0348 (18)	
H9BA	0.155460	0.446583	0.202724	0.042*	
H9BB	0.358888	0.435960	0.176956	0.042*	
C10B	0.4180 (19)	0.2929 (11)	0.2131 (2)	0.040 (2)	
H10C	0.315155	0.234480	0.227048	0.048*	
H10D	0.518178	0.223425	0.201255	0.048*	
C11B	0.5807 (19)	0.3881 (12)	0.2322 (2)	0.040 (2)	
H11C	0.480899	0.457543	0.244083	0.048*	
H11D	0.684051	0.446439	0.218283	0.048*	
C12B	0.743 (2)	0.3013 (11)	0.2545 (3)	0.046 (3)	
H12C	0.639419	0.240664	0.267950	0.055*	
H12D	0.845679	0.233823	0.242513	0.055*	
C13B	0.902 (3)	0.3955 (12)	0.2744 (3)	0.054 (3)	
H13C	0.799784	0.461581	0.286800	0.064*	
H13D	1.004162	0.457444	0.261043	0.064*	
C14B	1.065 (2)	0.3061 (13)	0.2960 (3)	0.056 (3)	
H14C	0.962078	0.246454	0.309691	0.067*	
H14D	1.162754	0.237694	0.283542	0.067*	
C15B	1.232 (3)	0.3997 (14)	0.3155 (3)	0.064 (4)	
H15C	1.134613	0.469594	0.327604	0.077*	
H15D	1.337595	0.457688	0.301799	0.077*	
C16B	1.392 (3)	0.3100 (15)	0.3377 (4)	0.068 (4)	
H16C	1.286969	0.252074	0.351459	0.082*	
H16D	1.489971	0.240101	0.325639	0.082*	
C17B	1.558 (3)	0.4038 (14)	0.3570 (3)	0.069 (4)	
H17C	1.458790	0.475483	0.368448	0.082*	
H17D	1.664076	0.459935	0.343077	0.082*	
C18B	1.721 (3)	0.3159 (16)	0.3807 (4)	0.077 (4)	
H18C	1.614009	0.258535	0.394380	0.093*	
H18D	1.821044	0.245277	0.369189	0.093*	
C19B	1.874 (3)	0.4044 (16)	0.3992 (4)	0.075 (4)	
H19C	1.774431	0.472867	0.411380	0.090*	
H19D	1.978561	0.463942	0.385667	0.090*	
C20B	2.037 (3)	0.314 (2)	0.4216 (5)	0.098 (6)	
H20C	1.932315	0.259883	0.435952	0.118*	
H20D	2.127849	0.240942	0.409475	0.118*	
C21B	2.219 (4)	0.405 (2)	0.4406 (4)	0.107 (6)	
H21D	2.133384	0.483177	0.451476	0.129*	
H21E	2.338536	0.448541	0.426818	0.129*	
H21F	2.300394	0.341123	0.455478	0.129*	

### Atomic displacement parameters (Å^2^)

**Table:**

	*U*^11^	*U*^22^	*U*^33^	*U*^12^	*U*^13^	*U*^23^
N1A	0.040 (5)	0.028 (4)	0.049 (4)	−0.003 (4)	0.001 (4)	0.000 (3)
C1A	0.018 (4)	0.055 (6)	0.031 (4)	0.005 (5)	0.000 (3)	0.003 (4)
S1A	0.0325 (13)	0.0268 (11)	0.0605 (14)	−0.0012 (11)	−0.0109 (10)	−0.0003 (11)
C2A	0.032 (5)	0.024 (5)	0.074 (7)	0.003 (4)	0.007 (5)	−0.006 (5)
S2A	0.0344 (14)	0.0278 (11)	0.0710 (15)	−0.0035 (12)	−0.0150 (11)	0.0016 (12)
C3A	0.015 (4)	0.034 (5)	0.055 (5)	−0.003 (4)	−0.004 (4)	0.001 (4)
C4A	0.028 (5)	0.024 (4)	0.055 (5)	−0.003 (4)	−0.005 (4)	−0.003 (4)
C5A	0.031 (5)	0.029 (5)	0.062 (6)	0.002 (5)	0.011 (5)	−0.008 (4)
C6A	0.036 (6)	0.020 (4)	0.056 (5)	0.008 (4)	0.000 (4)	−0.005 (4)
C7A	0.031 (6)	0.035 (5)	0.060 (6)	0.006 (5)	−0.004 (4)	−0.002 (5)
C8A	0.040 (6)	0.028 (5)	0.053 (5)	0.009 (5)	0.002 (4)	0.003 (4)
C9A	0.034 (6)	0.040 (6)	0.063 (6)	0.020 (5)	−0.003 (5)	0.008 (5)
C10A	0.052 (7)	0.032 (6)	0.059 (6)	0.016 (5)	0.007 (5)	0.007 (4)
C11A	0.043 (7)	0.041 (6)	0.066 (7)	0.026 (6)	0.011 (5)	0.015 (5)
C12A	0.067 (8)	0.042 (7)	0.070 (7)	0.032 (7)	0.009 (7)	0.016 (5)
C13A	0.070 (9)	0.052 (7)	0.064 (7)	0.039 (7)	0.019 (6)	0.022 (6)
C14A	0.089 (11)	0.042 (7)	0.061 (7)	0.027 (7)	0.015 (7)	0.014 (5)
C15A	0.082 (11)	0.054 (7)	0.072 (8)	0.037 (8)	0.034 (8)	0.023 (7)
C16A	0.117 (14)	0.057 (9)	0.075 (9)	0.042 (10)	0.038 (10)	0.028 (7)
C17A	0.131 (16)	0.080 (11)	0.093 (10)	0.073 (11)	0.058 (9)	0.051 (8)
C18A	0.140 (18)	0.090 (12)	0.098 (11)	0.070 (12)	0.055 (9)	0.049 (8)
C19A	0.141 (16)	0.108 (14)	0.097 (11)	0.087 (13)	0.072 (9)	0.062 (9)
C20A	0.17 (2)	0.116 (18)	0.130 (17)	0.083 (16)	0.043 (12)	0.041 (12)
C21A	0.18 (2)	0.14 (2)	0.137 (15)	0.114 (17)	0.086 (12)	0.092 (13)
N1B	0.022 (4)	0.031 (4)	0.047 (4)	−0.013 (3)	0.000 (3)	0.004 (3)
C1B	0.025 (4)	0.032 (5)	0.046 (5)	−0.012 (4)	−0.010 (4)	0.009 (4)
S1B	0.0309 (12)	0.0278 (11)	0.0580 (13)	−0.0003 (10)	−0.0033 (10)	0.0027 (10)
C2B	0.021 (5)	0.029 (4)	0.052 (5)	−0.009 (4)	0.000 (4)	−0.004 (4)
S2B	0.0181 (10)	0.0287 (10)	0.0568 (12)	−0.0021 (9)	−0.0032 (9)	−0.0009 (10)
C3B	0.016 (5)	0.033 (5)	0.066 (6)	−0.001 (4)	−0.014 (4)	−0.006 (4)
C4B	0.021 (5)	0.039 (6)	0.064 (7)	0.001 (4)	−0.002 (4)	−0.003 (4)
C5B	0.021 (4)	0.023 (4)	0.037 (4)	−0.002 (4)	−0.007 (3)	0.001 (3)
C6B	0.017 (4)	0.027 (4)	0.056 (5)	0.001 (4)	0.000 (4)	0.003 (4)
C7B	0.012 (4)	0.022 (4)	0.056 (5)	−0.008 (4)	−0.001 (3)	−0.006 (4)
C8B	0.025 (5)	0.022 (4)	0.071 (7)	0.007 (4)	0.009 (4)	0.003 (4)
C9B	0.020 (4)	0.024 (4)	0.060 (5)	−0.001 (4)	0.001 (4)	−0.002 (4)
C10B	0.028 (5)	0.028 (5)	0.064 (6)	0.009 (4)	0.003 (4)	0.000 (4)
C11B	0.030 (5)	0.033 (5)	0.056 (6)	0.003 (5)	0.007 (4)	0.005 (4)
C12B	0.036 (6)	0.026 (5)	0.075 (7)	0.013 (5)	0.013 (5)	0.014 (5)
C13B	0.060 (8)	0.030 (6)	0.072 (7)	0.010 (6)	0.019 (6)	0.015 (5)
C14B	0.044 (7)	0.035 (6)	0.090 (9)	0.022 (6)	0.013 (6)	0.025 (6)
C15B	0.067 (9)	0.048 (7)	0.077 (8)	0.032 (7)	0.020 (7)	0.028 (6)
C16B	0.067 (9)	0.045 (7)	0.093 (9)	0.040 (7)	0.024 (8)	0.039 (7)
C17B	0.091 (11)	0.041 (7)	0.074 (8)	0.025 (7)	0.026 (7)	0.021 (6)
C18B	0.076 (10)	0.048 (7)	0.108 (10)	0.033 (7)	0.039 (7)	0.039 (6)
C19B	0.080 (10)	0.052 (8)	0.094 (9)	0.037 (7)	0.035 (7)	0.030 (6)
C20B	0.076 (11)	0.092 (12)	0.126 (13)	0.057 (9)	0.046 (7)	0.072 (10)
C21B	0.115 (15)	0.105 (15)	0.102 (12)	0.055 (11)	0.046 (9)	0.033 (10)

### Geometric parameters (Å, º)

**Table:**

N1A—C3A	1.289 (14)	N1B—C3B	1.316 (14)
N1A—C2A	1.340 (14)	N1B—C2B	1.417 (11)
C1A—C2A	1.363 (15)	C1B—C2B	1.348 (14)
C1A—S1A	1.711 (11)	C1B—S1B	1.763 (11)
C1A—H1A	0.9500	C1B—H1B	0.9500
S1A—C3A	1.758 (10)	S1B—C3B	1.744 (11)
C2A—H2A	0.9500	C2B—H2B	0.9500
S2A—C3A	1.733 (10)	S2B—C3B	1.740 (11)
S2A—C4A	1.799 (10)	S2B—C4B	1.799 (11)
C4A—C5A	1.543 (14)	C4B—C5B	1.557 (13)
C4A—H4AA	0.9900	C4B—H4BA	0.9900
C4A—H4AB	0.9900	C4B—H4BB	0.9900
C5A—C6A	1.484 (15)	C5B—C6B	1.516 (12)
C5A—H5AA	0.9900	C5B—H5BA	0.9900
C5A—H5AB	0.9900	C5B—H5BB	0.9900
C6A—C7A	1.539 (14)	C6B—C7B	1.521 (12)
C6A—H6AA	0.9900	C6B—H6BA	0.9900
C6A—H6AB	0.9900	C6B—H6BB	0.9900
C7A—C8A	1.507 (15)	C7B—C8B	1.511 (13)
C7A—H7AA	0.9900	C7B—H7BA	0.9900
C7A—H7AB	0.9900	C7B—H7BB	0.9900
C8A—C9A	1.516 (13)	C8B—C9B	1.508 (14)
C8A—H8AA	0.9900	C8B—H8BA	0.9900
C8A—H8AB	0.9900	C8B—H8BB	0.9900
C9A—C10A	1.520 (17)	C9B—C10B	1.524 (14)
C9A—H9AA	0.9900	C9B—H9BA	0.9900
C9A—H9AB	0.9900	C9B—H9BB	0.9900
C10A—C11A	1.533 (15)	C10B—C11B	1.497 (15)
C10A—H10A	0.9900	C10B—H10C	0.9900
C10A—H10B	0.9900	C10B—H10D	0.9900
C11A—C12A	1.49 (2)	C11B—C12B	1.540 (14)
C11A—H11A	0.9900	C11B—H11C	0.9900
C11A—H11B	0.9900	C11B—H11D	0.9900
C12A—C13A	1.556 (16)	C12B—C13B	1.500 (19)
C12A—H12A	0.9900	C12B—H12C	0.9900
C12A—H12B	0.9900	C12B—H12D	0.9900
C13A—C14A	1.45 (2)	C13B—C14B	1.533 (16)
C13A—H13A	0.9900	C13B—H13C	0.9900
C13A—H13B	0.9900	C13B—H13D	0.9900
C14A—C15A	1.570 (19)	C14B—C15B	1.52 (2)
C14A—H14A	0.9900	C14B—H14C	0.9900
C14A—H14B	0.9900	C14B—H14D	0.9900
C15A—C16A	1.45 (3)	C15B—C16B	1.539 (17)
C15A—H15A	0.9900	C15B—H15C	0.9900
C15A—H15B	0.9900	C15B—H15D	0.9900
C16A—C17A	1.59 (2)	C16B—C17B	1.51 (2)
C16A—H16A	0.9900	C16B—H16C	0.9900
C16A—H16B	0.9900	C16B—H16D	0.9900
C17A—C18A	1.49 (3)	C17B—C18B	1.59 (2)
C17A—H17A	0.9900	C17B—H17C	0.9900
C17A—H17B	0.9900	C17B—H17D	0.9900
C18A—C19A	1.60 (3)	C18B—C19B	1.42 (3)
C18A—H18A	0.9900	C18B—H18C	0.9900
C18A—H18B	0.9900	C18B—H18D	0.9900
C19A—C20A	1.44 (4)	C19B—C20B	1.56 (2)
C19A—H19A	0.9900	C19B—H19C	0.9900
C19A—H19B	0.9900	C19B—H19D	0.9900
C20A—C21A	1.59 (3)	C20B—C21B	1.54 (3)
C20A—H20A	0.9900	C20B—H20C	0.9900
C20A—H20B	0.9900	C20B—H20D	0.9900
C21A—H21A	0.9800	C21B—H21D	0.9800
C21A—H21B	0.9800	C21B—H21E	0.9800
C21A—H21C	0.9800	C21B—H21F	0.9800
			
C3A—N1A—C2A	112.0 (9)	C3B—N1B—C2B	110.7 (9)
C2A—C1A—S1A	110.3 (7)	C2B—C1B—S1B	107.8 (7)
C2A—C1A—H1A	124.9	C2B—C1B—H1B	126.1
S1A—C1A—H1A	124.9	S1B—C1B—H1B	126.1
C1A—S1A—C3A	88.2 (5)	C3B—S1B—C1B	90.8 (5)
N1A—C2A—C1A	115.6 (10)	C1B—C2B—N1B	117.1 (9)
N1A—C2A—H2A	122.2	C1B—C2B—H2B	121.5
C1A—C2A—H2A	122.2	N1B—C2B—H2B	121.5
C3A—S2A—C4A	100.4 (5)	C3B—S2B—C4B	101.3 (5)
N1A—C3A—S2A	129.8 (8)	N1B—C3B—S2B	124.6 (8)
N1A—C3A—S1A	113.9 (8)	N1B—C3B—S1B	113.4 (8)
S2A—C3A—S1A	116.3 (6)	S2B—C3B—S1B	122.0 (6)
C5A—C4A—S2A	104.4 (7)	C5B—C4B—S2B	110.4 (8)
C5A—C4A—H4AA	110.9	C5B—C4B—H4BA	109.6
S2A—C4A—H4AA	110.9	S2B—C4B—H4BA	109.6
C5A—C4A—H4AB	110.9	C5B—C4B—H4BB	109.6
S2A—C4A—H4AB	110.9	S2B—C4B—H4BB	109.6
H4AA—C4A—H4AB	108.9	H4BA—C4B—H4BB	108.1
C6A—C5A—C4A	112.9 (8)	C6B—C5B—C4B	110.5 (8)
C6A—C5A—H5AA	109.0	C6B—C5B—H5BA	109.6
C4A—C5A—H5AA	109.0	C4B—C5B—H5BA	109.6
C6A—C5A—H5AB	109.0	C6B—C5B—H5BB	109.6
C4A—C5A—H5AB	109.0	C4B—C5B—H5BB	109.6
H5AA—C5A—H5AB	107.8	H5BA—C5B—H5BB	108.1
C5A—C6A—C7A	110.5 (9)	C5B—C6B—C7B	111.3 (8)
C5A—C6A—H6AA	109.6	C5B—C6B—H6BA	109.4
C7A—C6A—H6AA	109.6	C7B—C6B—H6BA	109.4
C5A—C6A—H6AB	109.6	C5B—C6B—H6BB	109.4
C7A—C6A—H6AB	109.6	C7B—C6B—H6BB	109.4
H6AA—C6A—H6AB	108.1	H6BA—C6B—H6BB	108.0
C8A—C7A—C6A	113.9 (8)	C8B—C7B—C6B	114.0 (8)
C8A—C7A—H7AA	108.8	C8B—C7B—H7BA	108.7
C6A—C7A—H7AA	108.8	C6B—C7B—H7BA	108.7
C8A—C7A—H7AB	108.8	C8B—C7B—H7BB	108.7
C6A—C7A—H7AB	108.8	C6B—C7B—H7BB	108.7
H7AA—C7A—H7AB	107.7	H7BA—C7B—H7BB	107.6
C7A—C8A—C9A	113.5 (9)	C9B—C8B—C7B	113.4 (8)
C7A—C8A—H8AA	108.9	C9B—C8B—H8BA	108.9
C9A—C8A—H8AA	108.9	C7B—C8B—H8BA	108.9
C7A—C8A—H8AB	108.9	C9B—C8B—H8BB	108.9
C9A—C8A—H8AB	108.9	C7B—C8B—H8BB	108.9
H8AA—C8A—H8AB	107.7	H8BA—C8B—H8BB	107.7
C8A—C9A—C10A	113.6 (9)	C8B—C9B—C10B	113.7 (8)
C8A—C9A—H9AA	108.8	C8B—C9B—H9BA	108.8
C10A—C9A—H9AA	108.8	C10B—C9B—H9BA	108.8
C8A—C9A—H9AB	108.8	C8B—C9B—H9BB	108.8
C10A—C9A—H9AB	108.8	C10B—C9B—H9BB	108.8
H9AA—C9A—H9AB	107.7	H9BA—C9B—H9BB	107.7
C9A—C10A—C11A	113.3 (9)	C11B—C10B—C9B	114.1 (8)
C9A—C10A—H10A	108.9	C11B—C10B—H10C	108.7
C11A—C10A—H10A	108.9	C9B—C10B—H10C	108.7
C9A—C10A—H10B	108.9	C11B—C10B—H10D	108.7
C11A—C10A—H10B	108.9	C9B—C10B—H10D	108.7
H10A—C10A—H10B	107.7	H10C—C10B—H10D	107.6
C12A—C11A—C10A	113.2 (10)	C10B—C11B—C12B	113.6 (9)
C12A—C11A—H11A	108.9	C10B—C11B—H11C	108.9
C10A—C11A—H11A	108.9	C12B—C11B—H11C	108.9
C12A—C11A—H11B	108.9	C10B—C11B—H11D	108.9
C10A—C11A—H11B	108.9	C12B—C11B—H11D	108.9
H11A—C11A—H11B	107.7	H11C—C11B—H11D	107.7
C11A—C12A—C13A	113.9 (10)	C13B—C12B—C11B	114.2 (8)
C11A—C12A—H12A	108.8	C13B—C12B—H12C	108.7
C13A—C12A—H12A	108.8	C11B—C12B—H12C	108.7
C11A—C12A—H12B	108.8	C13B—C12B—H12D	108.7
C13A—C12A—H12B	108.8	C11B—C12B—H12D	108.7
H12A—C12A—H12B	107.7	H12C—C12B—H12D	107.6
C14A—C13A—C12A	114.0 (11)	C12B—C13B—C14B	113.0 (9)
C14A—C13A—H13A	108.7	C12B—C13B—H13C	109.0
C12A—C13A—H13A	108.7	C14B—C13B—H13C	109.0
C14A—C13A—H13B	108.7	C12B—C13B—H13D	109.0
C12A—C13A—H13B	108.7	C14B—C13B—H13D	109.0
H13A—C13A—H13B	107.6	H13C—C13B—H13D	107.8
C13A—C14A—C15A	113.6 (11)	C15B—C14B—C13B	113.6 (10)
C13A—C14A—H14A	108.8	C15B—C14B—H14C	108.8
C15A—C14A—H14A	108.8	C13B—C14B—H14C	108.8
C13A—C14A—H14B	108.8	C15B—C14B—H14D	108.8
C15A—C14A—H14B	108.8	C13B—C14B—H14D	108.8
H14A—C14A—H14B	107.7	H14C—C14B—H14D	107.7
C16A—C15A—C14A	113.1 (12)	C14B—C15B—C16B	113.6 (11)
C16A—C15A—H15A	109.0	C14B—C15B—H15C	108.8
C14A—C15A—H15A	109.0	C16B—C15B—H15C	108.8
C16A—C15A—H15B	109.0	C14B—C15B—H15D	108.8
C14A—C15A—H15B	109.0	C16B—C15B—H15D	108.8
H15A—C15A—H15B	107.8	H15C—C15B—H15D	107.7
C15A—C16A—C17A	113.5 (13)	C17B—C16B—C15B	113.2 (11)
C15A—C16A—H16A	108.9	C17B—C16B—H16C	108.9
C17A—C16A—H16A	108.9	C15B—C16B—H16C	108.9
C15A—C16A—H16B	108.9	C17B—C16B—H16D	108.9
C17A—C16A—H16B	108.9	C15B—C16B—H16D	108.9
H16A—C16A—H16B	107.7	H16C—C16B—H16D	107.8
C18A—C17A—C16A	114.1 (15)	C16B—C17B—C18B	114.8 (11)
C18A—C17A—H17A	108.7	C16B—C17B—H17C	108.6
C16A—C17A—H17A	108.7	C18B—C17B—H17C	108.6
C18A—C17A—H17B	108.7	C16B—C17B—H17D	108.6
C16A—C17A—H17B	108.7	C18B—C17B—H17D	108.6
H17A—C17A—H17B	107.6	H17C—C17B—H17D	107.5
C17A—C18A—C19A	116.4 (16)	C19B—C18B—C17B	114.9 (12)
C17A—C18A—H18A	108.2	C19B—C18B—H18C	108.5
C19A—C18A—H18A	108.2	C17B—C18B—H18C	108.5
C17A—C18A—H18B	108.2	C19B—C18B—H18D	108.5
C19A—C18A—H18B	108.2	C17B—C18B—H18D	108.5
H18A—C18A—H18B	107.3	H18C—C18B—H18D	107.5
C20A—C19A—C18A	115.9 (18)	C18B—C19B—C20B	113.5 (13)
C20A—C19A—H19A	108.3	C18B—C19B—H19C	108.9
C18A—C19A—H19A	108.3	C20B—C19B—H19C	108.9
C20A—C19A—H19B	108.3	C18B—C19B—H19D	108.9
C18A—C19A—H19B	108.3	C20B—C19B—H19D	108.9
H19A—C19A—H19B	107.4	H19C—C19B—H19D	107.7
C19A—C20A—C21A	119 (2)	C21B—C20B—C19B	115.3 (15)
C19A—C20A—H20A	107.7	C21B—C20B—H20C	108.5
C21A—C20A—H20A	107.7	C19B—C20B—H20C	108.5
C19A—C20A—H20B	107.7	C21B—C20B—H20D	108.5
C21A—C20A—H20B	107.7	C19B—C20B—H20D	108.5
H20A—C20A—H20B	107.1	H20C—C20B—H20D	107.5
C20A—C21A—H21A	109.5	C20B—C21B—H21D	109.5
C20A—C21A—H21B	109.5	C20B—C21B—H21E	109.5
H21A—C21A—H21B	109.5	H21D—C21B—H21E	109.5
C20A—C21A—H21C	109.5	C20B—C21B—H21F	109.5
H21A—C21A—H21C	109.5	H21D—C21B—H21F	109.5
H21B—C21A—H21C	109.5	H21E—C21B—H21F	109.5
			
C2A—C1A—S1A—C3A	1.3 (8)	C2B—C1B—S1B—C3B	−3.9 (7)
C3A—N1A—C2A—C1A	−0.4 (14)	S1B—C1B—C2B—N1B	5.6 (10)
S1A—C1A—C2A—N1A	−0.8 (12)	C3B—N1B—C2B—C1B	−4.7 (12)
C2A—N1A—C3A—S2A	−179.7 (8)	C2B—N1B—C3B—S2B	179.6 (7)
C2A—N1A—C3A—S1A	1.5 (12)	C2B—N1B—C3B—S1B	1.3 (10)
C4A—S2A—C3A—N1A	5.9 (12)	C4B—S2B—C3B—N1B	26.9 (10)
C4A—S2A—C3A—S1A	−175.3 (6)	C4B—S2B—C3B—S1B	−154.9 (6)
C1A—S1A—C3A—N1A	−1.6 (9)	C1B—S1B—C3B—N1B	1.5 (8)
C1A—S1A—C3A—S2A	179.3 (6)	C1B—S1B—C3B—S2B	−176.9 (6)
C3A—S2A—C4A—C5A	177.9 (7)	C3B—S2B—C4B—C5B	70.6 (8)
S2A—C4A—C5A—C6A	−179.7 (7)	S2B—C4B—C5B—C6B	178.1 (7)
C4A—C5A—C6A—C7A	179.7 (9)	C4B—C5B—C6B—C7B	−177.7 (8)
C5A—C6A—C7A—C8A	−178.1 (9)	C5B—C6B—C7B—C8B	179.6 (8)
C6A—C7A—C8A—C9A	178.3 (9)	C6B—C7B—C8B—C9B	−179.7 (8)
C7A—C8A—C9A—C10A	179.7 (10)	C7B—C8B—C9B—C10B	179.5 (8)
C8A—C9A—C10A—C11A	−178.9 (9)	C8B—C9B—C10B—C11B	179.8 (9)
C9A—C10A—C11A—C12A	−178.8 (11)	C9B—C10B—C11B—C12B	180.0 (8)
C10A—C11A—C12A—C13A	−178.4 (10)	C10B—C11B—C12B—C13B	−178.4 (9)
C11A—C12A—C13A—C14A	−178.7 (12)	C11B—C12B—C13B—C14B	−178.9 (9)
C12A—C13A—C14A—C15A	−179.4 (11)	C12B—C13B—C14B—C15B	178.2 (10)
C13A—C14A—C15A—C16A	−178.6 (13)	C13B—C14B—C15B—C16B	178.8 (10)
C14A—C15A—C16A—C17A	−179.3 (12)	C14B—C15B—C16B—C17B	180.0 (11)
C15A—C16A—C17A—C18A	−179.4 (14)	C15B—C16B—C17B—C18B	178.6 (11)
C16A—C17A—C18A—C19A	−178.2 (13)	C16B—C17B—C18B—C19B	−179.1 (13)
C17A—C18A—C19A—C20A	−179.0 (19)	C17B—C18B—C19B—C20B	−178.3 (12)
C18A—C19A—C20A—C21A	178.8 (16)	C18B—C19B—C20B—C21B	176.1 (14)
